# Supplementary material for: Divisively normalized neuronal processing of uncertain visual feedback for visuomotor learning
Source: Commun Biol. 2023 Dec 20;6:1286. doi: 10.1038/s42003-023-05578-4 (PMC10733368; doi:10.1038/s42003-023-05578-4)
Supplement: Supplementary file 2 — Supplemenatry Information [file 42003_2023_5578_MOESM2_ESM.pdf]

Supplementary Information

Divisively normalized neuronal processing of uncertain visual feedback for visuomotor learning

Yuto Makino[1,2], Takuji Hayashi[1], Daichi Nozaki[1*]

[1] Division of Physical and Health Education, Graduate School of Education, The University of Tokyo, Tokyo, Japan

[2] Japan Society for the Promotion of Science, Tokyo, Japan

*Corresponding author

Supplementary Figure 1 and Figure 2

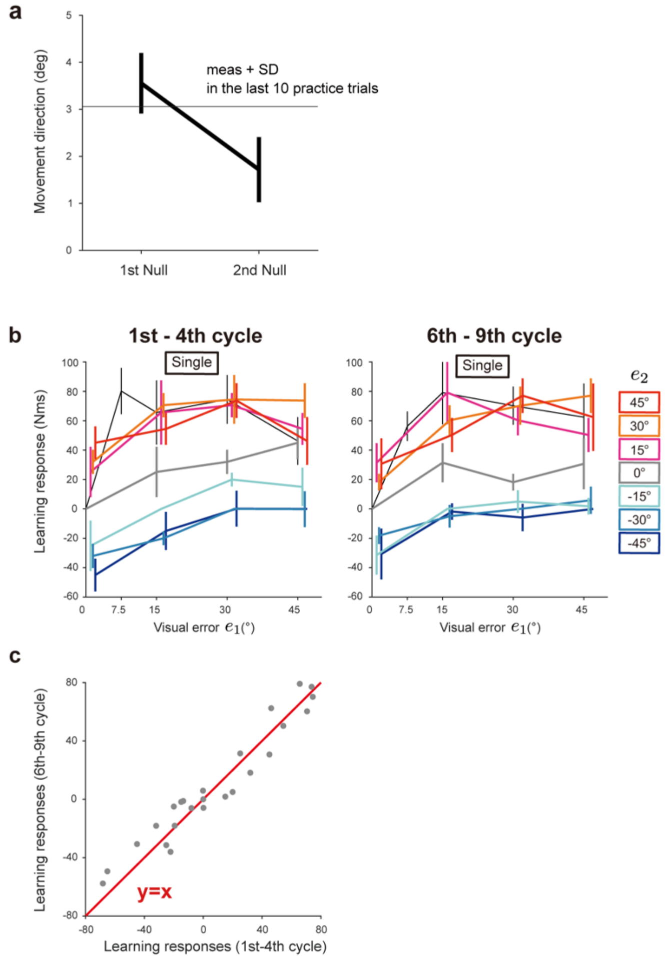

Supplementary Figure 1. (a) The aftereffect for the single perturbation conditions in the 1st and 2nd null washout trials after the probe trial. The aftereffects decreased below the mean+s.d. level of the last 10 practice trials, indicating that the washout was almost complete. (b) Learning responses for the earlier experimental phase (1st – 4th cycles) and for the later phase (6th-9th cycles). (c) There was no systematic change in the learning responses between these two experimental phases. Therefore, even if the learning effect was not completely eliminated, it did not accumulate to affect the results.

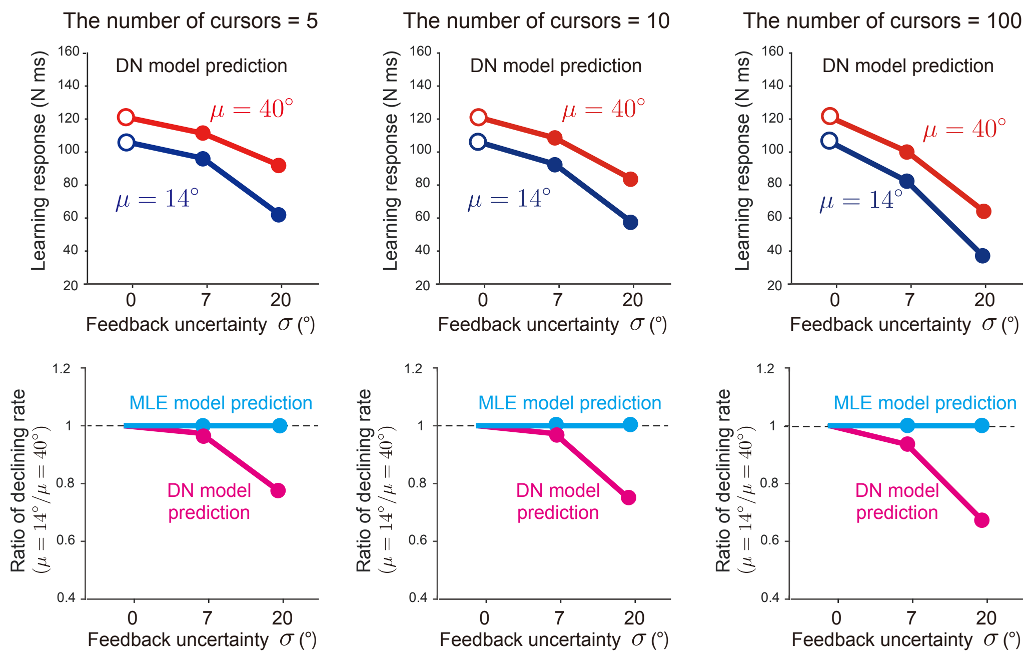

Supplementary Figure 2. Predictions of the DN model when the number of cursors is increased from 5 to 10 and 100. Even when the number is increased, the DN model continues to predict that learning responses decrease with the visual feedback uncertainty (σ) and that the ratio between the two mean perturbation sizes (μ = 14° / μ = 40°) of the declining rate of the learning response relative to that for σ = 0° decreases with the visual feedback uncertainty. The ordinary MLE model does not predict such an effect.
