# Supplementary material for: Divisively normalized neuronal processing of uncertain visual feedback for visuomotor learning
Source: Commun Biol. 2023 Dec 20;6:1286. doi: 10.1038/s42003-023-05578-4 (PMC10733368; doi:10.1038/s42003-023-05578-4)
Supplement: Supplementary file 3 — Description of Additional Supplementary Files [file 42003_2023_5578_MOESM3_ESM.docx]

Description of Additional Supplementary Files

**File name:** Supplementary Data 1

**Description**: The source data behind the graphs in the paper
